# A Systematic Review of Intervention Studies Examining Nutritional and Herbal Therapies for Mild Cognitive Impairment and Dementia Using Neuroimaging Methods: Study Characteristics and Intervention Efficacy

**DOI:** 10.1155/2017/6083629

**Published:** 2017-02-19

**Authors:** Genevieve Z. Steiner, Danielle C. Mathersul, Freya MacMillan, David A. Camfield, Nerida L. Klupp, Sai W. Seto, Yong Huang, Mark I. Hohenberg, Dennis H. Chang

**Affiliations:** ^1^National Institute of Complementary Medicine (NICM), Western Sydney University, Penrith, NSW 2751, Australia; ^2^School of Science and Health, Western Sydney University, Penrith, NSW 2751, Australia; ^3^War Related Illness and Injury Study Center, Veterans Affairs Palo Alto Health Care System, Palo Alto, CA 94304, USA; ^4^School of Medicine, Stanford University, Stanford, CA 94305, USA; ^5^Department of Psychology, University of Pennsylvania, Philadelphia, PA 19104, USA; ^6^School of Psychology and Illawarra Health & Medical Research Institute (IHMRI), University of Wollongong, Wollongong, NSW 2252, Australia; ^7^Centre for Human Psychopharmacology, Swinburne University of Technology, Hawthorne, VIC 3122, Australia; ^8^School of Traditional Chinese Medicine, Southern Medical University, Guangzhou 510515, China; ^9^School of Medicine, Western Sydney University, Penrith, NSW 2751, Australia; ^10^Department of Medicine, Campbelltown Hospital, South Western Sydney Area Health Service, Campbelltown, NSW 2560, Australia

## Abstract

Neuroimaging facilitates the assessment of complementary medicines (CMs) by providing a noninvasive insight into their mechanisms of action in the human brain. This is important for identifying the potential treatment options for target disease cohorts with complex pathophysiologies. The aim of this systematic review was to evaluate study characteristics, intervention efficacy, and the structural and functional neuroimaging methods used in research assessing nutritional and herbal medicines for mild cognitive impairment (MCI) and dementia. Six databases were searched for articles reporting on CMs, dementia, and neuroimaging methods. Data were extracted from 21/2,742 eligible full text articles and risk of bias was assessed. Nine studies examined people with Alzheimer's disease, 7 MCI, 4 vascular dementia, and 1 all-cause dementia. Ten studies tested herbal medicines, 8 vitamins and supplements, and 3 nootropics. Ten studies used electroencephalography (EEG), 5 structural magnetic resonance imaging (MRI), 2 functional MRI (fMRI), 3 cerebral blood flow (CBF), 1 single photon emission tomography (SPECT), and 1 positron emission tomography (PET). Four studies had a low risk of bias, with the majority consistently demonstrating inadequate reporting on randomisation, allocation concealment, blinding, and power calculations. A narrative synthesis approach was assumed due to heterogeneity in study methods, interventions, target cohorts, and quality. Eleven key recommendations are suggested to advance future work in this area.

## 1. Introduction

Dementia is a syndrome comprising over 100 diseases and is characterised by a decline in cognition that interferes with function and independence [1]. Over 46.8 million people worldwide have a diagnosis of dementia [2], and currently there is no cure. Dementia has a heterogeneous pathophysiology, with multiple mechanisms thought to play a role in the various types. For example, there are several hypotheses on the pathogenesis of Alzheimer's disease (AD) alone (the most common type of dementia, making up approximately 60–80% of all cases [3]) including the amyloid-beta peptide hypothesis, the inflammation hypothesis, the tau hypothesis, and the cholinergic hypothesis [4]. Oxidative stress, hypoxia, calcium imbalance, abnormal metal accumulation, amyloid-beta peptide accumulation within mitochondria, and brain-specific insulin signalling deficiencies are all thought to play a role in the complex pathophysiology of AD [5, 6]. Because of this, first-line single target pharmacological therapies for AD, acetylcholinesterase (AChE) inhibitors (e.g., donepezil) and N-methyl-D-aspartate (NMDA) receptor antagonists (e.g., memantine), are not particularly effective, boosting cognitive function in the early disease stages only, and are unable to slow or stop the disease progression [7, 8].

In the absence of effective pharmaceutical options for dementia, complementary medicines (CMs) have been thoroughly explored. Randomised-controlled trials (RCTs) have been conducted on a range of CMs for dementia, cognitive decline, and mild cognitive impairment (MCI), with many studies currently ongoing. This research has largely focused on nutritional and herbal medicine interventions (e.g., resveratrol, anthocyanins, fish oil, vitamins B and E,* Ginkgo biloba*,* Curcuma longa*,* Bacopa monnieri*, and multi-herb formulas such as Sailuotong [SLT]), dietary interventions (e.g., ketogenic and Mediterranean diets), mind-body interventions (e.g., mindfulness, yoga, tai chi, and other types of physical activity), and manual therapies (e.g., acupuncture), and has yielded mixed results due to a range of methodological inconsistencies. Therapies that show potential as adjunct treatments for dementia, or prevention methods, should be thoroughly investigated with the most rigorous and objective measures to reduce sources of bias.

Neuroimaging techniques can provide an objective, precise, and noninvasive measure of neuronal function and are particularly useful in the assessment of complementary therapies for dementia. Popular functional techniques applied in CM research include electroencephalography (EEG), functional magnetic resonance imaging (fMRI), positron emission tomography (PET), magnetoencephalography (MEG), single photon emission computed tomography (SPECT), and functional near-infrared spectroscopy (fNIRS). Structural magnetic resonance imaging (MRI) and diffusion tensor imaging (DTI) can also be used to assess changes in morphology following longer interventions. As detailed in Table 1, depending on study characteristics such as the sample's degree of cognitive impairment, intervention type and duration, neurocognitive function of interest, and reasons for using neuroimaging, these methodologies have a range of advantages and limitations that should be considered carefully before a specific technique is applied in a CM dementia research study.

Neuroimaging, in particular functional neuroimaging, can be utilised in dementia CM research as a sensitive measure of neurocognition, with the capacity to record changes that cannot otherwise be detected by standard pen-and-paper neuropsychological tests. This is useful given the small effect sizes often reported in CM research, particularly acute studies, and that any proposed intervention for cognitive decline is effectively fighting an uphill battle against neurodegenerative pathophysiology. Furthermore, some techniques can be used to explore the mechanisms of action of a therapy, which is particularly useful in psychopharmacological studies (e.g., nutritional and herbal medicines).

The aim of this systematic review was three-fold: (1) provide a comparison and critical evaluation of the characteristics of studies assessing nutritional and herbal medicines for MCI and dementia; (2) evaluate their use of structural and functional neuroimaging methods; (3) summarise intervention efficacy. The Preferred Reporting Items for Systematic Reviews and Meta-Analyses (PRISMA) statement [9] was followed during the planning, conduct, and writing of this review.

## 2. Methods

### 2.1. Eligibility Criteria

Several initial scoping reviews were conducted to determine the eligibility criteria and review scope. Eligibility criteria were determined in line with the PICO principles for systematic reviews [10]:

- *Population.* People with cognitive decline, MCI, or dementia
- *Intervention.* Chronic CM treatment
- *Comparisons.* Placebo or control group
- *Outcome.* Structural or functional neuroimaging method

Peer-reviewed studies were included if they reported a herbal or nutritional intervention for MCI or dementia and either structural or functional neuroimaging as an outcome measure. It should be noted that the search strategy was intentionally kept broad and also included both mind-body (e.g., yoga) and manual treatments (e.g., acupuncture); due to the large volume of results, only studies assessing nutritional and herbal interventions were included. Reviews, commentaries, conference proceedings, editorials, preclinical (in vitro and in vivo), and acute clinical studies were excluded, as were studies that were not published in English, or when the full text could not be retrieved.

### 2.2. Search Strategy

The research team and an experienced librarian reviewed the search strategy before systematic searching commenced. Six databases were searched for studies published in peer-reviewed journals. Abstracts were retrieved from PubMed, ScienceDirect, Web of Science, ProQuest, Scopus, and PsycINFO ranging from databases' dates of inception to August 28, 2016. A full list of keywords and an example of the search strategy for the Scopus database are detailed in Supplementary Material available online at https://doi.org/10.1155/2017/6083629 (Table S1). Similar searches were carried out in the other five databases, with only minor modifications to permit changes in the use of searching symbols. Reference lists of key articles were also searched for other eligible studies.

### 2.3. Data Extraction and Appraisal

One reviewer examined the titles and abstracts of each article. If there was any doubt regarding the eligibility of an article, the full-text was retrieved for clarification. Articles deemed eligible by one reviewer were further assessed by two other independent reviewers to ensure inclusion criteria were met. Any disagreements were resolved by reviewing the full papers and a subsequent discussion.

Study characteristics were extracted from each full-text article. Data extracted included title, authors, publication date, aim, study type, disease focus, study population characteristics, number of participants (target cohort and controls), age (mean/median and SD), gender ratio, participant recruitment, diagnostic criteria, neuroimaging technique and analysis method, neuropsychological test battery, definition and dosage of CM, length of intervention, follow-up, and findings.

An assessment of methodological risk of bias in individual studies was conducted. A 10-item scale was constructed to suit the relevancy of studies in this review. The scale was informed by the Cochrane Handbook [11] and the Quality Checklist for Healthcare Intervention Studies [12] (detailed in Table 2) to capture major sources of bias including selection bias, internal and external validity bias, reporting bias, and statistical bias. For each study, the following elements were assessed: random sequence generation, allocation concealment, sampling, blinding, intervention description, neuroimaging methodology, validity and reliability of outcome measures, selective reporting, adverse events, and statistical power. Each of the 10 items on the scale were rated as yes (scored as 1), no, or unable to determine (both scored as 0), allowing higher scores to indicate a lower risk of bias. Studies with total scores ≥ 9 were considered to have a low risk of bias.

As there was substantial heterogeneity across included studies (in neuroimaging methods, intervention types, and study quality), quantitative analyses (i.e., meta-analysis) were not appropriate. Consequently, this review assumed a qualitative approach with a narrative analysis. The characteristics of each study were extracted, and data were described using a narrative synthesis approach.

## 3. Results

### 3.1. Study Selection

**Figure 1:**
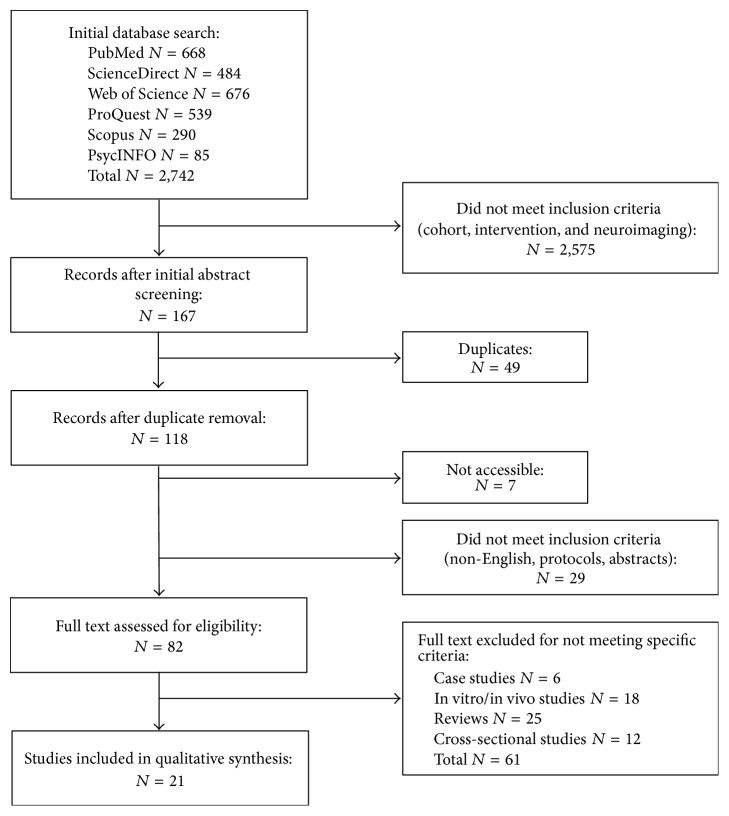
Flow diagram illustrating the study selection process.

Figure 1 illustrates the study selection process. Twenty-one studies [13–33] met the inclusion criteria for review. Three studies [16–18] reported results from the same RCT; the other 18 papers contained unique studies. Ten studies assessed herbal medicines [13, 19, 21, 23, 26, 29–33], 8 focused on vitamins and supplements [15–18, 22, 25, 27, 28], and 3 were on nootropics [14, 20, 23] (i.e., cognitive enhancers).

### 3.2. Study Characteristics

Table 3 details a summary of the characteristics of the 21 studies including aims, setting, population, intervention type and duration, neuroimaging methods and measures, efficacy of intervention, adverse events, adherence, and retention. Most (*n* = 15) studies included 1 intervention and 1 control group [13–20, 22, 24, 29–33], 1 study had 3 parallel arms [28], and five studies had no control group [21, 23, 25, 26, 34]. Four studies were carried out in China [13, 31–33], 3 each in Japan [23, 26, 30] and the United Kingdom [16–18], 2 each in Italy [27, 28] and Germany [19, 22], and 1 each in the United States [14], Austria [20], Sweden [25], Greece [29], Romania [24], and Korea [21]. One study was a multisite RCT carried out in Belgium, France, Germany, Italy, the Netherlands, and Spain [15]. Three studies were published in the 1990s [19, 20, 26], 5 studies were published between 2000 and 2004 [14, 25, 27, 28, 30], and the other 13 were published after 2010 [13, 15–18, 21–24, 29, 31–33].

#### 3.2.1. Participants

Across all the included studies (taking into account the 3 studies on the same RCT [16–18]), the total sample size was *N* = 1,055 (476 males, 569 females, 1 study with 10 participants did not specify sex [26]; mean age = 70.9,* SD* = 6.8 years), with individual studies ranging from 8 to 179 participants, and 3 studies with less than 20 participants [21, 23, 26]. Sample size was determined with* a priori* power calculation in 3 studies [15, 18, 20], all of which achieved target sample size.

Nine studies tested 399 participants with AD [13, 15, 19, 21, 23, 25–28], 7 studies examined 319 participants with MCI [16, 17, 22, 23, 29, 31, 32], 4 studies analysed 156 participants with vascular dementia (VaD) [14, 24, 25, 33], 1 study explored 112 participants with unspecified dementia (all cause) [20], 1 study examined 9 participants with mixed-type dementia (combined AD and vascular pathologies) [25], and 1 study included 60 age-matched controls [28]. Twenty studies [13–16, 18–33] measured global cognition at baseline with the mini mental state examination (MMSE: mean score = 22.0,* SD* = 2.5).

#### 3.2.2. Recruitment

Four studies recruited from memory clinics [14, 15, 22, 29], 3 from the community [16–18], 2 from both hospitals and the community [31, 32], 2 from hospital outpatients [25, 30], 2 from hospital inpatients [13, 33], 1 from a medical centre [21], 1 from a nursing home [20], 1 from both outpatient clinics and the community [34], and 1 from a university clinic [23], and 5 did not specify a recruitment location [19, 24, 26–28].

#### 3.2.3. Intervention Design

All studies examined chronic administration with treatment duration ranging from 4 weeks [24, 25] to 2 years [16–18], with most chronic studies (*n* = 8) assessing the effects of a 12-week intervention [13, 19, 21, 26, 30–33]. Ten studies tested herbal interventions [13, 19, 21, 23, 26, 29–33], 8 assessed vitamins (B or E) [16–18, 25, 27, 28] or supplements [15, 22], and 3 tested nootropics [14, 20, 24]. Across all studies, 18 administered an oral intervention, of which 13 were in the form of a tablet/capsule [14, 16–22, 27, 28, 31–33], 3 as a granular or powder extract [13, 23, 30], 1 as a drink [15], and 2 with no details of the preparation method (traditional Chinese medicine [26] and Crocus [29]). One of those studies was a multidomain intervention with omega-3 fatty acid supplementation, aerobic training, and cognitive stimulation [22]. One study gave intramuscular injections [25] and 1 intravenous infusions [24].

#### 3.2.4. Neuroimaging Techniques

Ten studies used EEG [15, 19–21, 24, 26–30], 5 used MRI [14, 16–18, 22], 2 used fMRI [31, 32], 1 used SPECT [23] and another PET [13], and 3 studies measured CBF [25, 26, 33]. One CBF study employed xenon 133 inhalation and high resolution scintillation detectors (Cortexplorer®) [25], 1 used transcranial Doppler (TCD) [33], and the other used stable xenon CT; that study also recorded EEG [26]. One other study combined methods: EEG and MRI [29].

A range of analyses were conducted for EEG, MRI, and fMRI studies. For the EEG studies, 1 examined functional connectivity using phase lag index [15], 2 studies assessed relative power with quantitative EEG (qEEG) from an eyes closed resting state condition [21, 24], 1 study examined theta/alpha ratio [19], and 6 studies assessed P300 ERP component amplitudes and latencies from an auditory oddball task [20, 26–30], 1 of which also analysed N200 [29], and another also employed a 3-minute vigilance task and assessed absolute and relative power [20]. For the MRI studies, 1 examined whole brain volume and subcortical and periventricular hyperintensities [14], 1 regional volumetric changes [29], 2 examined regional grey matter volume [16, 22], and 2 examined whole brain atrophy [17, 18]. Of the 2 fMRI studies, both assessed blood oxygenation level dependent (BOLD) responses, 1 with an episodic memory encoding task [31], and another with an n-back task [32].

#### 3.2.5. Measures of Cognition

A variety of neuropsychological measures were used to assess cognition. The most common were the MMSE (*n* = 20) [13–18, 20–33], Alzheimer's Dementia Assessment Scale-cognitive subscale (ADAS-cog; *n* = 4) [13, 24, 27, 28], Hopkins Verbal Learning Test (HVLT-R; *n* = 4) [16–18], Auditory Verbal Learning Test (*n* = 3) [22, 31, 32], Rey Auditory Verbal Learning Test (*n* = 1) [15], California Verbal Learning Test (*n* = 3) [14, 31, 32], Stroop Test (*n* = 3) [22, 31, 32], Trail Making Test (*n* = 3) [14, 15, 22], Clinical Dementia Rating-Sum of Boxes (CDR-SOB; *n* = 3) [16–18], and Category Fluency (*n* = 3) [16–18]. Please refer to Table 3 for other neuropsychological tests used.

#### 3.2.6. Compliance, Withdrawals, and Adverse Events

Nine studies reported on compliance [15–18, 22–24, 27, 28], 14 reported on withdrawals (loss to follow-up) [13–18, 20, 22–24, 27, 28, 31, 32], and 11 reported on adverse events [13, 16–18, 20, 22, 23, 26, 27, 31, 32]. Information on the reporting of compliance, withdrawals, and adverse events is summarised in Table 3.

### 3.3. Risk of Bias within and across Studies

Table 4 details the results for the risk of bias assessment (refer to Table 2 for items assessed). Although 13 studies were randomised [13–18, 20, 24, 27, 28, 31–33], only 5 studies detailed how the randomisation procedure was conducted [15–18, 28], and 4 of those studies reported specific information on the allocation concealment [15–18]. Participant characteristics including how the diagnosis of cognitive decline, MCI, or dementia was made or confirmed, and inclusion and exclusion criteria were described in 16 studies [13–18, 20–25, 27, 31–33]. Seven studies reported on blinding of participants, intervention deliverers, and researchers collecting data [15–18, 27, 31, 32]. Only 12 out of the 21 studies provided sufficient information on the intervention to allow replication [13–18, 20, 23, 25, 27, 28, 33], and only 12 described neuroimaging methodologies and analyses sufficiently to allow replication [13–18, 22, 23, 25, 28, 31, 32]. Thirteen studies used appropriate, valid, and reliable outcome measures [13–18, 20, 22–24, 28, 31, 32]. The majority of studies did not selectively report [13–33]. Adverse events were reported in 12 studies [15–18, 20–23, 26, 27, 31, 32], and only 3 studies reported a power calculation, all of which were sufficiently powered to detect an effect [15, 18, 20].

#### 3.3.1. Intervention Efficacy in Low Risk of Bias Studies

Four of the 21 studies were reported particularly well and demonstrated a low risk of bias (scoring ≥ 9) [15–18]. Three of those studies were reporting on findings from the same randomised, double-blind, placebo-controlled trial (VITACOG) [16–18] investigating the effects of 2 years of high dose vitamin B treatment for people with MCI, and the other was a randomised, double-blind, placebo-controlled 24-week international multisite clinical trial [15] on Souvenaid® for AD. All 4 studies incorporated a relatively comprehensive neuropsychological test battery, rather than just a simple global measure of cognition (e.g., ADAS-cog, MMSE). One of those studies reported on EEG network connectivity [15], and the other 3 reported structural MRI: regional grey matter volume [16] and whole brain atrophy [17, 18]. One study found a reduction in EEG beta network integrity in the placebo, but not the intervention group, indicating counteraction of network decline after 24 weeks of 125 mL/day Souvenaid in people with AD [15]. The other 3 studies showed a reduction in regional grey matter and whole brain atrophy after 2 years treatment with high dose vitamin B (0.8 mg/day folic acid, 20 mg/day vitamin B6, 0.5 mg/day vitamin B12) for people with MCI, compared to placebo [16–18].

Three of the 4 studies reported associations between cognitive test scores and neuroimaging outcome measures [15, 16, 18]. In 1 study, an association between EEG beta activity and memory performance (*z*-score across NTB; see Table 3) was reported at midpoint in the Souvenaid group only [15]. An association between rate of atrophy and both final MMSE scores and baseline Telephone Interview of Cognitive Status-Modified (TICS-M) scores was reported in one of the high dose vitamin B studies [18]. There was also an association between increased grey matter loss and lower MMSE and CDR-SOB scores and poorer delayed recall and category fluency performance [16] in another of the vitamin B studies.

### 3.4. Efficacy on Neuroimaging Measures across All Studies

Eighteen studies reported positive neuroimaging findings associated with CM treatment [13, 15–26, 29–33] (8 MCI studies, 6 AD studies, 3 VaD studies, and 1 all-cause dementia study) and three reported negative findings [14, 27, 28] (2 AD studies and 1 VaD study). The key patterns of results are outlined below; for more detailed information on results not reported in the review body, please refer to Table 3.

Out of the 6 studies that assessed auditory oddball P300 ERP component amplitudes and latencies, 4 reported reduced P300 latencies [20, 26, 29, 30] and 2 reported increased P300 amplitudes [29, 30] after CM treatment (12 weeks of traditional Chinese medicine versus no comparison group [26]; 8 weeks of 60 mg/day nicergoline* cf.* placebo [20]; 12 weeks of 7.5 g/day Choto-san extract [TJ-47] versus no treatment [30]; 52 weeks of Crocus extract versus waitlist [29]). Two other studies [27, 28] reported similar changes in the control condition: both reported increased P300 amplitudes (26 weeks of 5 mg/day donepezil [27, 28] or 1.5 mg/day rivastigmine [28]), and 1 reported reduced P300 latencies (26 weeks of 5 mg/day donepezil) [27]. Those two studies also showed a decrease in P300 amplitude and an increase in latency following 26 weeks of 2000 IU/day vitamin E [27, 28]. Theta was significantly reduced in the theta/alpha quotient after 12-weeks treatment with 80 mg/day standardised ginkgo biloba extract (EGb761) in one study [19], and another study reported decreased beta network EEG in the placebo but not the intervention group (24 weeks of 125 mL/day Souvenaid) [15].

One MRI study showed significantly increased whole brain volume after 26 weeks of the target multimodal intervention (see Table 3 for details) and reduced volume for the control group [22], and another study showed no difference in whole brain volume between treatment (52 weeks of 1 g/day citicoline) and placebo [14]. One fMRI study reported both increased BOLD response in the right putamen and reduced BOLD in the right middle temporal gyrus when participants completed an episodic memory encoding task after 1.2 g/day Bushen for 12 weeks [31].

One CBF study reported an increase in white matter CBF with stable xenon CT after 12 weeks of a traditional Chinese medicine [26], and one TCD CBF study reported increased blood flow velocity to the middle and anterior cerebral arteries after 12 weeks of 19.2 mg/day EGb 761 standardised ginkgo extract and 75 mg/day aspirin [33].

### 3.5. Efficacy on Cognition across All Studies

Across all studies, 13 reported positive effects on cognition [13, 19–21, 23–26, 29–33], 4 studies reported negative results [14, 22, 27, 28], and 4 did not report on cognition findings alone [15–18]. As detailed above in Section 3.4, the key patterns of results for the commonly used neuropsychological tests are detailed below, with further information available in Table 3. Two studies [13, 24] reported improvements (a reduction) in ADAS-cog scores in the intervention group (12 weeks of 10 g/day fuzhisan [13]; 4 weeks of 10 mL/day Cerebrolysin [24]), and 1 in the control group (26 weeks of 10 mg/day donepezil [27]), and another showed a significant deterioration in ADAS-cog scores following treatment in the CM arm (26 weeks of 2000 IU/day Vitamin E) but noted improvements in the other two parallel arms (5 mg/day donepezil and 1.5 mg/day rivastigmine) [28].

Five studies [20, 26, 30–32] reported significantly improved MMSE scores after treatment (12 weeks of a traditional Chinese medicine [26]; 8 weeks of 60 mg/day nicergoline [20]; 12 weeks of 22.5 g/day TJ-47 Choto-san extract [30]; 12 weeks of 1.2 g/day bushen [31]; 12 weeks of 3/day Congrongyizhi capsules [32]) and another showed a trend towards improved MMSE scores following 8 weeks of 7.5 g/day of toki-shakuyaku-san powder [23]. One study reported no changes in cognition following 6 months of a multimodal intervention [22].

### 3.6. Associations between Neuroimaging and Cognitive Measures

Six of 21 studies reported associations between measures of cognition and neuroimaging markers [15–18, 31, 32]. One study showed a relationship between activation in the right putamen during an episodic memory task and Stroop performance, and reduced middle temporal gyrus deactivation with AVLT performance [31]. Another study showed that greater posterior cingulate cortex deactivation was associated with improved MMSE and digit span scores [32].

Four studies did not report neuropsychological test battery findings alone as they had already been published previously [15–18]. For example, one of those studies reported an association between memory performance and EEG beta band activity at the midpoint of the 24-week Souvenaid trial (125 mL/day) [15]. Please see Table 3 for more detailed information on studies reporting associations between clinical and neuroimaging findings.

## 4. Discussion

This systematic review summarised and critically appraised intervention studies that incorporated neuroimaging outcome measures to assess nutritional and herbal medicines for MCI and dementia. The majority of studies focused on participants with AD [13, 15, 19, 21, 23, 25–28] or MCI [16, 17, 22, 23, 29, 31, 32], utilised a herbal medicine [13, 19, 21, 23, 26, 29–33], a 12-week long intervention [13, 19, 21, 26, 30–33], and incorporated EEG [15, 19–21, 24, 26–30] or structural MRI [14, 16–18, 22] as a neuroimaging technique. All but 3 studies [14, 27, 28] reported positive neuroimaging results following CM treatment [13, 15–26, 29–33], despite most (*n* = 17) studies having a high risk of bias, scoring ≤ 6 out of 10 on the risk of bias assessment [13, 14, 19–33]. Given the importance of using neuroimaging markers in the assessment of endpoints for clinical trials in dementia [35], particularly with a move towards preclinical disease phases [36], and the viable role that CMs can play as potential treatments, it is imperative that the rigour and quality of CM dementia studies using neuroimaging techniques is improved. This discussion will now focus separately on the three aims of this review: (1) study characteristics; (2) methodologies; and (3) intervention efficacy. To address risk of bias, an additional discussion on study quality has also been included. In light of the findings from this systematic review, a series of key recommendations for improving future work in this area is detailed in Box 1.

### 4.1. Study Characteristics

#### 4.1.1. Participants

The majority of studies reported information on how a diagnosis of MCI or dementia was made or confirmed and included sufficient inclusion and exclusion criteria to allow replication [13–18, 20–25, 27, 31–33]; one study did not detail cognitive status of the control group [30]. Important demographic information, such as years of education, a factor known to significantly increase the risk of dementia [37], was missing from other studies [19, 28]. In order to meaningfully assess the efficacy of an intervention, it is essential that the tested cohort is as homogeneous as possible. This can be done by closely following guidelines stipulating the most up-to-date diagnostic criteria for MCI [38], dementia (relative to the type; e.g., McKhann et al. [39]), and subjective cognitive complaints [40], and by carefully recording and reporting all relevant participant demographics and baseline characteristics. Care must also be taken to match participant characteristics between active and control groups, with one study not detailing information on the cognitive status of the control group, making comparison impossible [30].

#### 4.1.2. Study Setting

The majority of studies recruited from memory clinics [14, 15, 22, 29], hospitals [13, 23, 33], the community [16–18], or a combination of those settings [31, 32]. However, 5 studies did not report the recruitment setting [19, 24, 26–28]. The recruitment setting for dementia studies has been shown to dramatically influence the participant characteristics and health outcomes. For example, participants with MCI recruited from a memory clinic have been shown to have an annual conversion rate to dementia that is 10% higher than participants recruited from the community [41]. Thus, future work in this field should ensure that the recruitment setting is carefully considered in study design and reported adequately in the published results.

#### 4.1.3. Intervention

The majority of studies tested a Chinese herbal medicine [13, 19, 21, 23, 26, 30–33] or a vitamin [16–18, 25, 27, 28] intervention, with most using a tablet or capsule for oral administration [14, 16–22, 27, 28, 31–33]. Only just over half the studies reported enough detail for the intervention to be replicated [13–18, 20, 23, 25, 27, 28, 33]. The main difficulty here was that, for herbal medicines, standardisation did not occur [26, 30], or the details were not supplied. For the latter, this included missing information on the particular standardised formula used (e.g., EGb 761 for* Ginkgo biloba*), missing information on dose or dosing regimen, and/or inadequate information on commercially available extracts (e.g., brand/manufacturer) [19, 21, 22, 26, 29–32]. Quality control and quality assurance (Good Manufacturing Practice [GMP]) is required for psychopharmacological research, and the absence of complete information on intervention formulation makes results near impossible to replicate. This problem is further compounded when multi-herb formulas are used, as a greater degree of preclinical work is required to develop standard operating procedures (SOPs) for extraction methods, and to optimise ratios of individual constituents. It should also be noted that treatment duration varied substantially between studies from 4 weeks [24, 25] to 2 years [16–18], adding further complexity to comparisons between studies.

#### 4.1.4. Study Design

Although the majority of studies included a control group [13–20, 22, 24, 27–33], four studies did not [21, 23, 25, 26], rendering a high risk of bias. A control group, such as a placebo, should always be incorporated to establish whether a true relationship between the treatment and outcome actually exists. In the context of herbal medicine research, appropriate placebos are often difficult to establish because they need to match the active treatment on taste, smell, look, and feel. Herbal medicines can be pungent and have a distinctive taste so additional care needs to be taken when matching to a placebo [42].

### 4.2. Methodology

#### 4.2.1. Structural and Functional Neuroimaging Methods

Most studies incorporated functional neuroimaging methods [13, 15, 19–21, 23–28, 30–33], largely EEG [15, 19–21, 24, 26–30]. There were large differences in the tasks and analytic methods described in these studies, but the majority of EEG papers assessed auditory oddball P300 ERP component amplitudes and latencies [20, 26–30]. The P300 has been widely explored in ERP literature and has been associated with a range of cognitive processes including memory [43], the orienting of attention [44], decision-making [45], and expectancy [46, 47]. The studies assessing P300 in this review largely reported baseline-to-peak quantification methods (when quantification was described at all), despite this being an ineffective approach for disentangling the multiple subcomponents comprised within the monolithic P300 peak (i.e., P3a, P3b, Novelty P3, and Slow Wave) that represent a range of cognitive processes [48]. Given that effect sizes from CMs can be small [49], and that interventions may affect various cognitive domains, it is imperative that optimal analytic methods are employed to maximise the chance of detecting an effect. Alternative component quantification methods, such as Principal Components Analysis (PCA), should be adopted for future CM ERP studies [50].

Neuroimaging data acquisition, pre- and postprocessing pipelines, and analyses were adequately reported in only 12 of 21 studies [13–18, 22, 23, 25, 28, 31, 32]. There was insufficient information on how the data were collected (e.g., recording parameters, task details including length of resting state condition, and stimulus delivery) [19–21, 24, 26, 30, 33], inadequate reporting of pre- and postprocessing techniques that are in line with widely accepted best practice (e.g., artefact rejection) [21, 29], and missing data quantification details (e.g., Fast Fourier Transformation [FFT] parameters, quantification of P300) [24, 26]. Given the potential limitations of some neuroimaging techniques (as outlined in Table 1), it is imperative that future work describes all data acquisition, processing, and analytic techniques to ensure that variability in results between studies can be adequately accounted for.

Although the majority of studies reported positive results [13, 15–26, 29–33], as noted above, the quality of reporting in most of these studies was relatively poor, indicating a high risk of bias. The results and conclusions from those studies should be viewed with a degree of caution. Given that functional neuroimaging methods are often more sensitive than standard pen-and-paper tests, it is even more important that high quality data, analyses, and interpretations are reported.

#### 4.2.2. Measures of Cognition

The majority of studies utilised the MMSE [13–18, 20–28, 30–33], ADAS-cog [13, 24, 27, 28], and tested verbal learning [14–18, 22, 31, 32]. Similar to the neuroimaging results, most studies reported positive effects on cognition [13, 19–21, 23–26, 30–33], even though the risk of bias assessment indicated that only 13 studies used appropriate outcome measures [13–18, 20, 22–24, 28, 31, 32]. For example, it has been argued that the MMSE is not appropriate for cognitive assessments in people with MCI due to its low sensitivity (18%) in that cohort [51]. However, all but 1 [17] of the 7 MCI studies included here reported MMSE scores. These shortcomings make it challenging to meaningfully interpret the efficacy on cognition of the CMs reviewed here. The 4 studies that scored a low risk of bias utilised comprehensive neuropsychological test batteries [15–18] and did not report on the efficacy of these cognitive outcome measures as they had already been reported previously when the complete results of those RCTs were published elsewhere. Future work should also utilise a comprehensive neuropsychological test battery and use outcome measures that are appropriate clinical trial endpoints for the level of cognitive impairment of the target cohort [52].

### 4.3. Study Quality and Risk of Bias

The majority of studies assessed in this systematic review were at high risk of bias [13, 14, 19–33]. One of the most common (and significant) issues was that a power calculation was not reported in the majority of studies (Table 4). Most studies had a relatively small sample size and were consequently at risk of Type II error (false negative). The 3 studies that did conduct a power calculation all achieved their recruitment target [15, 18, 20]. Bias also came from a lack of reporting on how randomisation and allocation concealment were carried out. Most studies were randomised trials [13–18, 20, 24, 27, 28, 31–33]; however, only a small number of these actually reported on the randomisation procedure [15–18, 28] and an even smaller number on how allocation was concealed [15–18]. Randomisation allows for the distribution of participant characteristics to be left to chance. Without adequate randomisation, it cannot be assumed that the null hypothesis (that participant groups have been drawn from the same population) is true [53]; this jeopardises internal validity. In relation to allocation concealment, given that most studies utilised an oral intervention, there is no reason that similar future work should not report how allocation was concealed and who was blinded. It must be acknowledged that this is not always the case in some physical activity interventions [22], where allocation concealment can be challenging. A further source of bias came from the lack of reporting of adverse events, which was done by only 12 studies [15–18, 20–23, 26, 27, 31, 32]. Future work should always report adverse events that may have been due to the intervention as it ensures the safety of participants.

### 4.4. Intervention Efficacy

The focus of the 4 high quality studies that scored a low risk of bias [15–18] was to report detailed analyses of neuroimaging secondary outcome measures. Of those four studies, 3 reported that 2-year treatment for MCI with high dose vitamin B (0.8 mg/day folic acid, 20 mg/day vitamin B6, and 0.5 mg/day vitamin B12) reduced whole brain and regional grey matter atrophy, compared to placebo [16–18], and 1 found that 24 weeks of 125 mL/day Souvenaid maintained EEG beta network integrity in people with AD, where this declined in the placebo group [15].

Three of those studies also reported an association between cognitive test scores and neuroimaging outcome measures [15, 16, 18]. It was found that lower MMSE, CDR-SOB, delayed recall, and category fluency scores were associated with accelerated grey matter loss in one of the high dose vitamin B studies [16]. Baseline TICS-M and final MMSE scores were associated with rate of atrophy in another high dose vitamin B study [18], and midpoint memory performance was associated with beta activity in the Souvenaid study [15]. In terms of clinical use, the above studies indicate that 2 years of high dose vitamin B or 6 months of 125 mL/day Souvenaid have potential clinical utility as an adjunct therapy for people with MCI or Alzheimer's disease, respectively.

### 4.5. Recommendations

This systematic review has identified a number of consistent shortcomings in CM neuroimaging research into cognitive decline. In an effort to improve the rigour and validity of this important and developing field, the authors suggest 11 key recommendations emerging from the 3 review aims that future work should adhere to. These are detailed in Box 1.

### 4.6. Strengths and Limitations

This systematic review focused on studies reporting a chronic intervention only. Acute studies may necessarily utilise a different range of neuroimaging methods than those reported here. For example, structural MRI is not appropriate for acute treatment administration as structural brain changes take longer than a few hours to be detected. Future research should systematically summarise and critically appraise acute CM studies [54, 55] to provide a more comprehensive overview of the field. Furthermore, the heterogeneity of the interventions and neuroimaging techniques employed made meta-analyses impossible here. Future work (with a different aim) could consider focusing on only one intervention or neuroimaging modality in order to quantify efficacy. It should also be noted that the authors of included studies were not contacted by the authors of this review.

This review not only focused on efficacy but also on summarising the characteristics of studies, intervention efficacy, and methods utilised. Particular consideration was given to identifying risks of bias. Neuroimaging and CM are a rapidly evolving area of research; thus the findings reported here highlight a number of significant strengths and weaknesses in this field that can be addressed in future work in an effort to improve the evidence base.

### 4.7. Conclusions

This systematic review summarised and critically appraised CM research on people with cognitive decline, MCI, or dementia that incorporated neuroimaging as an outcome measure. It was found that most studies focused on people with AD, utilised a herbal medicine intervention that was on average 12 weeks long, and used EEG or structural MRI as neuroimaging outcome measures. Nearly all studies reported positive results, despite the majority having a high risk of bias. The most common issues were a lack of reporting on randomisation, allocation concealment, blinding, and the lack of a power calculation. Eleven recommendations to improve future neuroimaging CM research on people with MCI and dementia have been highlighted in the recommendations box. The authors hope that the pragmatic approach taken to this systematic review will lead to an uptake of these recommendations and a subsequent increase in the quality of CM neuroimaging research on people with MCI or dementia.

## Supplementary Material

Table S1. Keywords and example search strategy used in Scopus.

## Figures and Tables

**Box 1:**
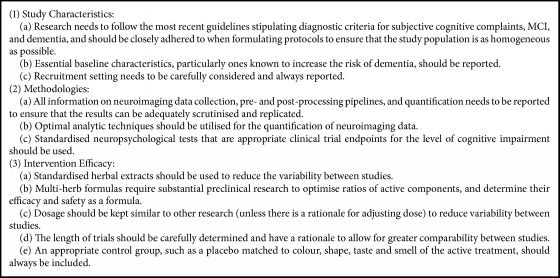
Recommendations for future chronic CM neuroimaging research on people with MCI or dementia.

**Table 1:** Neuroimaging technique, description, example quantification methods (by no means exhaustive), advantages, limitations, and relevance in CM studies on people with dementia.

Neuroimaging technique	Description	Quantification	Advantages	Limitations	Relevance in CM dementia research
EEG	Quantifies the electrical activity of the brain generated by electrical field potentials from excitatory and inhibitory neuronal activity.	Resting state EEG spectral activity (delta, theta, alpha, beta, gamma): power analyses and scalp-based functional connectivity measures (coherence, phase-lag). Event-related measures: ERPs, EPs, SST, and ERSP including ERS and ERD.	Very high temporal resolution, relatively inexpensive, noninvasive, portable options available.	Poor spatial resolution due to volume conduction.^*∗*^ Not as well-suited to investigations of subcortical dementia.	Captures subtle changes in cognitive and/or sensory function. Allows for the mechanisms of action of CM therapies to be explored. Noninvasive and portable options increase its usability in groups with more significant impairments. Suitable for acute and chronic studies.

fMRI	Measures changes in brain blood flow caused by neuronal activity.	Resting state: Region of interest functional connectivity approach. Event-related: BOLD response.	Good spatial resolution, particularly with high resolution scanners (e.g., 7 T).	Poor temporal resolution: the BOLD response lags by 1-2 s behind the actual neuronal activity. Claustrophobia and high-pitched noises can make scanning uncomfortable for participants.	Captures changes in cognitive and/or sensory function that can have their source localised in the brain.

SPECT	Quantifies changes in brain blood flow and metabolism. Nuclear gamma camera captures a gamma-emitting tracer being absorbed by brain tissue at the same rate as blood flow.	Regional CBF.	Relatively cheap compared to other functional imaging methods (e.g., PET, fMRI).	Administration of radioactive isotope (usually injection) and exposure to gamma radiation. Low spatial resolution (1 cm).	Useful in assessing interventions for dementia as SPECT can differentiate dementia pathologies (e.g., vascular dementia versus Alzheimer's disease). Allows for the mechanisms of action of CM therapies to be explored.

PET	Typically assesses regional brain glucose metabolism by detecting gamma rays emitted by a positron-emitting tracer.	Regional CBF.	Different novel isotopes allow distinction between Alzheimer's pathology and other dementias (PiB-PET).	Administration of radioactive isotope.	Different isotopes allow for tagging of different biochemical processes (e.g., FDG-PET for glucose uptake, or PiB-PET for amyloid imaging). Allows for the mechanisms of action of CM therapies to be explored.

MEG	Measures magnetic fields generated by the electrical activity of the brain.	Similar to EEG, resting state measures (delta, theta, alpha, beta, gamma band power) and event-related measures are available.	Very high temporal resolution, and better spatial resolution (i.e., more accurate) compared to EEG.	Detects only tangential components of current source, so primarily sensitive to activity within sulci.	Captures subtle changes in cognitive and/or sensory function. Allows for the mechanisms of action of CM therapies to be explored. Suitable for acute and chronic studies.

fNIRS	fNIRS captures changes in blood flow by detecting haemoglobin concentrations through the transmission and absorption of NIR light.	A range of measures are used, DOT or NIRI being popular forms of fNIR.	Noninvasive and portable.	Limitations when trying to measure activity in subcortical tissue.	CM intervention-associated changes in CBF can be ascertained. Mechanism of action can be explored due to the modulation of haemoglobin.

MRI	Structural MRI images the anatomy of the brain using magnetic fields, radio waves, and field gradients.	Most frequently used measures are voxel-based morphometry and ROI analyses.	Good spatial resolution, particularly with high resolution scanners (e.g., 7 T).	No functional information available.	Volumetric changes in brain regions (or whole brain) can be investigated. Any changes are best explored in chronic studies.

DTI	Measures diffusion of water in order to provide information on tissue microstructures so that white matter pathways within and between brain regions can be explored.	Tractography and tensor estimation.	Exploration of brain networks is becoming increasingly popular within the field. Better resolution with more angles (e.g., 61 direction scan).	No functional information available.	White matter integrity and structural network connectivity can be explored. Any changes are best explored in chronic studies.

*Note*. BOLD = blood oxygenation level dependent; CBF = cerebral blood flow; CM = complementary therapies; DOT = diffuse optical tomography; DTI = diffusion tensor imaging; EEG = electroencephalograph; EPs = evoked potentials; ERD = event-related de-synchronisation; ERPs = event-related potentials; ERSP = event-related spectral perturbation; ERS = event-related synchronisation; FDG-PET = fluorodeoxyglucose-positron emission tomography; MRI = magnetic resonance imaging; MEG = magnetoencephalograph; NIRI = near-infrared imaging; NIRS = near-infrared spectroscopy; PET = positron emission tomography; PiB-PET = Pittsburgh compound B-positron emission tomography; ROI = region of interest; SPECT = single photon emission computed tomography; SST = steady-state topography.

^*∗*^Can be addressed to a certain extent in connectivity analyses which partial out instantaneous zero-phase contributions.

**Table 2:** Risk of bias scale item descriptions.

Risk of bias item	Label	Description
1	Random sequence generation	Was the allocation sequence adequately generated?
2	Allocation concealment	Was allocation adequately concealed?
3	Participant characteristics	Are the characteristics of the participants included in the study clearly described (inclusion/exclusion criteria)?
4	Blinding of participants, personnel, and outcome assessors	Was knowledge of the allocated intervention adequately prevented during the study?
5	Intervention description	Is the intervention of interest sufficiently described to allow replication?
6	Neuroimaging methodology	Are the neuroimaging methods clearly described? Description should include data-acquisition parameters and pre- and postprocessing pipelines.
7	Outcome measurement validity and reliability	Were the outcome measures used accurate and appropriate (valid and reliable)?
8	Selective reporting	Were all outcome measures detailed in the methods reported in the results?
9	Adverse events	Have all important adverse events that may be a consequence of the intervention been reported?
10	Reporting of power calculation and attrition rate effect on power	Was a power calculation reported and was the study adequately powered to detect hypothesised relationships?

*Note*. Items rated as “yes” were scored as 1. Items rated as “no” or “unable to determine” were both scored as 0. Higher scores indicate a lower risk of bias.

**Table 3:** Summary of study characteristics, intervention, neuroimaging, and neuropsychological methodologies, efficacy, and study design.

Author (reference)	Aim, recruitment	Study population	Intervention and duration	Neuroimaging and neuropsychological measures	Efficacy on cognition, neuroimaging measures, any associations, adherence, retention, and adverse events	Study design
Bi et al. (2011) [13], China	Aim: to test the efficacy of fuzhisan (FZS) in people with AD. Recruitment location: hospital patients.	Mild to moderate AD (*N* = 25): randomised to FZS (7M : 5F, 72.50 ± 6.90 yrs, MMSE 19.58 ± 3.23, ADAS-Cog 33.58 ± 6.24, NPI 19.50 ± 6.24) or placebo (6M : 4F, 68.60 ± 6.35 yrs, MMSE 19.70 ± 3.30, ADAS-Cog 34.50 ± 5.80, NPI 19.80 ± 6.11).	Intervention: 1 × 10 g FZS (or placebo) per day, orally. Duration: 12 weeks. Free from over-the-counter medications for at least 2 weeks prior to study entry; free from psychotropic drugs for at least 4 weeks prior.	PET (30 min) NTB: MMSE, ADAS-cog, NPI.	Cognition: significant but small improvements in ADAS-Cog and NPI scores (versus no change in placebo). Neuroimaging: increased or stabilised rCMRglc across frontal, parietal, and temporal cortices, posterior cingulate gyrus, hippocampus, thalamus, cerebellum (versus decreased rCMRglc in placebo). Retention: total 3 withdrew: 1x FZS, 2x placebo (i.e., 88% completion rate). Adverse effects: 2x mild, transient side-effects (1x nausea, 1x constipation).	Randomised, double-blind, placebo-controlled pilot study.

Cohen et al. (2003) [14], USA	Aim: to investigate whether citicoline improves neurocognition & neuroimaging in people with VaD. Recruitment location: specialty care centre (memory & cognitive disorders clinic).	VaD (*N* = 30): randomised to citicoline (7M : 8F, 78.1 ± 5.8 yrs, MMSE 19.0 ± 4.7) or placebo (5M : 10F, 78.0 ± 5.1 yrs, MMSE 21.3 ± 2.9).	Intervention: 2 × 500 mg citicoline (or placebo) per day, orally. Duration: 12 months. Follow-up: 6 & 12 months.	MRI total brain volume MRI SH volume NTB: BNT, COWAT, CVLT, DRS, Grooved Pegboard Test, MMSE, RCFT, TMT, WAIS-R [block design, digit span & symbol, similarities, vocabulary], WMS-R [logical memory, visual reproduction].	Cognition: no difference between citicoline and placebo in neuropsychological performance (i.e., both groups significantly declined from baseline to both 6- and 12-month follow-up). Neuroimaging: no difference between citicoline and placebo in structural brain measures (i.e., both groups had significantly decreased total brain volume & increased SH from baseline to 12-month follow-up). Retention: total 9 withdrew prior to follow-up: 4x citicoline, 5x placebo.	Randomised, double-blind, placebo-controlled trial.

de Waal et al. (2014) [15], Belgium, France, Germany, Italy, The Netherlands, Spain	Aim: to investigate the effect of Souvenaid on brain-activity based networks in people with AD. Recruitment location: AD medical centres.	Mild AD (*N* = 179; drug-naïve): randomised to Souvenaid (45M : 41F, 74.1 ± 6.8 yrs, MMSE 25.1 ± 2.9) or placebo (47M : 46F, 72.5 ± 8.0 yrs, MMSE 25.4 ± 2.7).	Intervention: 1 × 125 mL drink (active Souvenaid or isocaloric control) per day. Duration: 24 weeks. Follow-up: 12 & 24 weeks.	EEG functional connectivity networks NTB: MMSE, COWAT, RAVLT, TMT, WMS-R [digit span, verbal paired associates].	Cognition: no relationship between EEG network and NTB memory performance. Association between beta activity and memory performance at midpoint in the treatment group only. Neuroimaging: decreased beta network EEG in the placebo but not Souvenaid group. *Suggests improved synaptic integrity and function and counteraction of network decline*. Retention: total 12 withdrew prior to follow-up; 14 excluded due to protocol deviations &/or <80% compliance: 4x Souvenaid, 10x placebo.	Randomised, double-blind, placebo-controlled, multisite trial.

Douaud et al. (2013) [16], Jernerén et al. (2015) [17] and Smith et al. (2010) [18], UK	Aim: (1) to investigate the effect of B-vitamin treatment on brain atrophy in people with MCI. (2) To investigate the effect of (a) plasma *ω*-3 fatty acid concentrations, (b) plasma tHCy concentrations, on B-vitamin treatment of brain atrophy in people with MCI. Recruitment location: community.	MCI (*N* = 156) [15]: randomised to B-vitamin (33M : 47F, 77 ± 5 yrs, MMSE 28.5 ± 1.5) or placebo (27M : 49F, 76 ± 4 yrs, MMSE 28.5 ± 1.5). MCI (*N* = 168) [19, 20]: randomised to B-vitamin (35M : 50F, 77.0 ± 5.2 yrs, MMSE 28.3 ± 1.8) or placebo (31M : 52F, 76.2 ± 4.5 yrs, MMSE 28.3 ± 1.5).	Intervention: high dose B-vitamin treatment (folic acid 0.8 mg/d, vit. B6 (pyridoxine HCl) 20 mg/d, vit. B12 (cyanocobalamin) 0.5 mg/d). 1x active or placebo tablet per day. Duration: 24 months.	MRI regional grey matter volume [15] MRI whole brain atrophy [19, 20] NTB: CDR-SOB, MMSE, HVLT-R (delayed recall), category fluency (animals).	Neuroimaging: significantly reduced brain atrophy (0.5% versus 3.7%) in posterior brain regions (bilateral hippocampus, parahippocampal gyrus, retrosplenial precuneus, lingual and fusiform gyrus, cerebellum). Rate of brain atrophy was significantly slower (by 29.6%) with B-vitamin treatment than placebo. This effect was even greater (53% lower atrophy) for individuals with high baseline tHcy, a risk factor for brain atrophy. This effect was also even greater (40% lower atrophy) for individuals with high baseline *ω*-3 fatty acid concentrations, a risk factor for brain atrophy. Adherence: >78% took at least 75% of the tablets; 81.4% (70/84 active, 66/83 placebo) determined biologically compliant via blood samples. Retention: total 20 withdrew (11x B-vit, 9x placebo). Total 15 lost to death or cancer (7x B-vit, 8x placebo). Further 8 lost or excluded for “miscellaneous” reasons (5x B-vit, 3x placebo). Adverse effects: no significant safety issues or group differences in adverse events.	Randomised, double-blind, placebo-controlled trial (VITACOG study).

Heo et al. (2016) [21], Korea	Aim: To investigate the effect of Korean red ginseng (KRG) on brain activity in people with AD. Recruitment location: medical centre.	AD (*N* = 14; 3M : 11F, 74.93 ± 7.63 yrs, K-MMSE 19.93 ± 4.80).	Intervention: 4.5 g/d KRG, orally (total powder capsule, 6-yr-old root; KT&G Corporation, Daedeok District, Korea; 8.54% Ginsenosides). Duration: 12 weeks.	qEEG (resting, eyes closed) NTB: MMSE, FAB	Cognition: significant improvement in cognitive function (FAB). Neuroimaging: relative alpha power increased in temporal regions for responders versus nonresponders. *Suggests increased frontal lobe function*.	Open, case series study.

Hofferberth (1994) [19], Germany	Aim: to investigate the effect of *Ginkgo biloba* special extract (GBE; EGb 761) treatment on brain activity in people with AD.	AD (*N* = 42): randomised to GBE (14M : 7F, 63.6 yrs) or placebo (14M : 7F, 63.6 yrs).	Intervention: 80 mg/d GBE (or placebo), orally. Duration: 3 months. Follow-up: 1, 2, and 3 months.	EEG (theta/alpha quotient) NTB: memory, attention, choice reaction time.	Cognition: significant improvement in cognitive function following 1 month and 2 months and maintained following 3 months of GBE. Significant improvement in choice reaction time following 1 month and maintained following 2 and 3 months of GBE. Neuroimaging: significant reduction in theta wave component of theta/alpha quotient following 1 month and maintained following 2 and 3 months of GBE.	Randomised, double-blind, placebo-controlled trial.

Köbe et al. (2015) [22], Germany	Aim: to investigate the combined effects of omega-3 fatty acids (FA), aerobic exercise, and cognitive stimulation on brain atrophy in people with MCI. Recruitment location: memory clinics.	MCI (*N* = 22): randomised to target intervention (9M : 4F, 70.0 ± 7.2 yrs, MMSE 28.5 ± 1.1) or control intervention (5M : 4F, 70.0 ± 5.2 yrs, MMSE 27.9 ± 1.7).	Intervention: both target and control groups received omega-3 FA (2.2 g/d; 4x oral capsules daily). Target intervention: aerobic training (cycle ergometer; 2 × 45 min/wk) + cognitive stimulation (AKTIVA: *Aktive Kognitive Stimulation-Vorbeugung im Alter* (active cognitive stimulation-prevention in the elderly); 1x individual + 12x group sessions, 90 min duration, plus daily home practice, beginning week 4). Control intervention: nonaerobic training (stretching & toning; 2 × 45 min/wk). Duration: 6 months.	MRI brain volume NTB: digit span, TMT, Stroop, AVLT, verbal fluency.	Cognition: no change in executive function, memory, sensorimotor speed, or attention. Neuroimaging: increased or reduced atrophy in GM volume for target versus control intervention (middle and superior frontal cortices, frontal pole, angular cortex, posterior cingulate cortex). Significant decrease in total homocysteine concentration. Retention/adherence: original *N* = 35; *n* = 13 discontinued due to time constraints or poor compliance. Adverse effects: None.	Randomised controlled trial

Matsuoka et al. (2012) [23], Japan	Aim: to investigate the effect of toki-shakuyaku-san (TSS) on rCBF in people with MCI or AD. Recruitment location: university clinic.	MCI/AD (*n* = 8; 3M : 5F, 77.8 ± 4.9 yrs, MMSE 23.4 ± 3.6).	Intervention: 7.5 g TSS, orally (powder). Duration: daily for 8 wks.	rCBF (SPECT) NTB: MMSE, NPI, PSMS.	Cognition: no change in MMSE scores (trend toward improved orientation to place). Neuroimaging: significant increase in rCBF in posterior cingulate. Retention/adherence: original *N* = 13; *n* = 5 discontinued due to poor compliance, change in location, or withdrawal. Adverse effects: none.	Open, case series study.

Muresanu et al. (2010) [24], Romania	Aim: to investigate persistence of the effects of cerebrolysin on cognition & qEEG in people with VaD.	VaD (*N* = 33): randomised (2 : 3 : 3) to cerebrolysin 10 mL (4M : 9F, 72.46 ± 2.80 yrs, MMSE 18.92 ± 1.32), cerebrolysin 30 mL (7M : 4F, 70.36 ± 3.62 yrs, MMSE 20.27 ± 1.92), or placebo (5M : 4F, 71.89 ± 3.52 yrs, MMSE 18.89 ± 1.81).	Intervention: 50 mL i.v. infusions of cerebrolysin (10 mL + 40 mL saline or 30 mL + 20 mL saline) or placebo (saline) 5 days/wk. Duration: 4 weeks. Follow-up: 12 weeks (±1).	qEEG, eyes closed resting state NTB: MMSE, ADAS-cog.	Cognition: significant improvement in cognitive performance maintained at follow-up. Neuroimaging: significant (dose-dependent) reduction in qEEG power ratio maintained at follow-up. Retention/adherence: original study *N* = 41; *n* = 8 lost to follow-up or due to poor compliance (i.e., receiving new drug treatment).	Open-label extension of a randomised, double-blind, placebo-controlled trial.

Nilsson et al. (2000) [25], Sweden	Aim: to investigate the effect of cobalamin (vitamin B12) treatment on brain function in people with a medical history of cognitive deterioration. Recruitment location: hospital outpatients.	Mild to severe dementia (*N* = 29 (VaD: *n* = 13; AD: *n* = 7, mixed *n* = 9); 15M : 14F, 78.9 ± 6.8 yrs; MMSE 9–23).	Intervention: intramuscular injection of hydroxycobalamin (vit. B12); 1 mg every second day, total 10x. Duration: 1 month.	rCBF (xenon 133 inhalation and cortexplorer with 254 scintillation detectors) NTB: MMSE, OBS.	Cognition: *N* = 15 classified as “clinically improved” (orientation to time and space, recent memory), though dementia severity did not change. *N* = 14 did not show any clinical improvements. Neuroimaging: *N* = 15 “clinically improved” had significant increase in general blood flow level. *N* = 14 slight trend towards a decrease in blood flow.	Open, case series study.

Oishi et al. (1998) [26], Japan	Aim: to investigate the effect of traditional Chinese medicine treatment on brain function in people with AD.	AD (*N* = 10; 65 ± 8 yrs; MMSE 16.0 ± 5.1).	Intervention: traditional Chinese medicine (astragalus root 8 g, *Prunella vulgaris* 3 g, pueraria root 9 g, *Lycii fructus* 8 g, cnidium rhizome 5 g, rhubarb 1 g, alisma rhizome 6 g, peach kernel 6 g, ginseng 3 g, oyster shell 8 g). Duration: 3 months.	ERP (auditory oddball P300) rCBF (stable xenon CT method) NTB: MMSE.	Cognition: significant improvement in MMSE scores (though still below normal range). Neuroimaging: significantly improved (shortened) P300 latency. Increased white matter CBF. Adverse effects: none.	Open, case series study.

Onofrj et al. (2002) [27], Italy	Aim: to test the effects of donepezil (DPZ) versus vitamin E on brain function in people with varying severities of AD.	Mild to severe AD (*N* = 60 completed): first divided into mild AD (group I) versus moderate-severe AD (group II) then randomised to treatment: group I DPZ (6M : 9F, 65.2 ± 1.8 yrs, MMSE 21.5 ± 0.4, ADAS-Cog 22.3 ± 1.0), Group I vit E (7M : 8F, 65.5 ± 1.7 yrs, MMSE 21.5 ± 0.6, ADAS-Cog 22.5 ± 0.9), Group II DPZ (6M : 9F, 66.7 ± 1.5 yrs, MMSE 11.5 ± 0.6, ADAS-Cog 44.5 ± 1.2), Group II vit E (8M : 7F, 66.5 ± 1.6 yrs, MMSE 11.6 ± 0.4, ADAS-Cog 43.5 ± 1.4).	Titration: 14 days, 5 mg/day DPZ or 1000 IU/day vit E, orally. Intervention: 10 mg/day DPZ or 2000 IU/day vit E, orally. Duration: 6 months.	ERP (P300 auditory oddball) NTB: MMSE, ADAS-cog, WAIS.	Cognition: DPZ: significant improvement in neuropsychological test performance, regardless of AD severity, though more pronounced for moderate-severe than mild AD. Vitamin E: severe deterioration of neuropsychological test performance, regardless of AD severity. Neuroimaging: DPZ: significantly reduced P300 latency, regardless of AD severity, though more pronounced for moderate-severe than mild AD. Vitamin E: significantly increased P300 latency, regardless of AD severity. Retention/adherence: total 7 withdrew during initial titration phase (6x DPZ (adverse effects), 1x Vit E (noncompliance)). Adverse effects: 6x DPZ (3x nausea or abdominal discomfort, 3x confused agitation).	Pseudo-randomised, double-blind, controlled trial.

Saletu et al. (1995) [20], Austria	Aim: to test the efficacy of nicergoline (NIC) in people with unspecified dementia (all-cause). Recruitment location: nursing home for seniors.	Mild to moderate dementia (*N* = 112, MMSE 13–25; equal distribution SDAT : MID (*n* = 56), equally randomised (*n* = 28) to placebo control (PLAC) or treatment (NIC)): SDAT/NIC: 5M : 23F, 78 ± 7 yrs; SDAT/PLAC: 7M : 21F, 77 ± 10 yrs; MID/NIC: 6M : 22F, 81 ± 7 yrs; MID/PLAC: 9M : 19F, 79 ± 7 yrs.	Intervention: 2 × 30 mg NIC (or placebo) per day, orally. Duration: 8 weeks NIC or placebo, 2-week washout period.	EEG mapping (3 min V-EEG) ERP (P300 auditory oddball) NTB: MMSE, SCAG, CGI.	Cognition: significant improvements in CGI, MMSE, and SCAG (versus pretreatment and placebo group). Neuroimaging: decreased relative power delta/theta and alpha-1, increased relative power alpha-2 and beta (right temporal to frontotemporal and left parietal and temporo-occipital regions) (versus opposite effects in placebo). *Suggests improved vigilance*. Acceleration of total centroid power spectrum (versus pre- and placebo). Shortened latency of P300 (versus pre- and placebo). *Suggests improved information processing*. Retention: total 14 withdrew: 4x SDAT/NIC, 4x SDAT/PLAC, 4x MID/NIC, 2x MID/PLAC. Responder to nonresponder ratio: SDAT/NIC 16 : 8 (i.e., 66.6% responders), SDAT/PLAC 8 : 16, MID/NIC 17 : 7 (i.e., 70.83% responders), MID/PLAC 7 : 19. Adverse effects: 9 of 48 NIC reported adverse effects (mild or marked insomnia (×2), mild itching, blocked nose, sweating, dry mouth, diarrhea, weight loss, constipation, moderate rigor).	Randomised, double-blind, placebo-controlled crossover trial.

Thomas et al. (2001) [28], Italy	Aim: to test the effects of donepezil (DPZ) versus vitamin E versus rivastigmine (Riv) on brain function in people with AD.	Mild to moderately severe AD (*N* = 60): randomised to double-blind treatment (DPZ (9M : 11F, 66.50 ± 9.19 yrs, MMSE 16.0 ± 0.5, ADAS-Cog 33.34 ± 2.70) or vit E (10M : 10F, 65.50 ± 10.61 yrs, MMSE 16.0 ± 0.5, ADAS-Cog 33.45 ± 2.60)), or open trial Riv (9M : 11F, 65.00 ± 8.49 yrs, MMSE 16.0 ± 0.5, ADAS-Cog 33.39 ± 2.70). Age-matched control group (*N* = 60): 25M : 35F, 67.50 ± 14.85 yrs, MMSE 29.0 ± 0.4, ADAS-Cog 14.25 ± 0.50.	Titration: 1 month, 5 mg/d DPZ, 2000 IU/d vit E, or 1.5 mg/d Riv, orally. Intervention: 10 mg/d DPZ or 2000 IU/d vit E, orally. Riv: 1x capsule 2x/d; month 2: total 3 mg/d, month 3: total 6 mg/d, month 4: total 9 mg/d, months 5 & 6: total 12 mg/d. Duration: 6 months. Follow-up: each month.	ERP (P300 auditory oddball) NTB: MMSE, ADAS-Cog, WAIS subscales.	Cognition: DPZ and Riv: significant improvement in neuropsychological test performance. Vitamin E: severe deterioration of neuropsychological test performance. Neuroimaging: DPZ and Riv: significant reduction in P300 latency (no difference between). Vitamin E: significantly increased P300 latency. P300 latency changes were significantly correlated with neuropsychological test scores. Retention/adherence: total 4 withdrew from Riv (3x nausea, 1x noncompliance). Total 2 excluded from vit E (no detectable P300). Adverse effects: 3x nausea.	Randomised three-arm trial with one open-label arm and two double-blind arms.

Tsolaki et al. (in press) [29], Greece	Aim: to test the effects of *Crocus sativus* L. (saffron) on brain function in people with aMCI. Recruitment location: outpatient memory & dementia clinic.	aMCI (*N* = 35): *Crocus* (5M : 12F, 71.47 ± 6.73 yrs, MMSE 27.41 ± 1.70, MoCA 22.91 ± 3.00) or wait-list control (4M : 14F, 69.72 ± 7.33 yrs, MMSE 27.89 ± 1.84, MoCA 22.81 ± 2.64).	Intervention: Crocus (no further information available). Follow-up: 12 months.	MRI (global maxima of case “a”; *n* = 8 subgroup) ERP (P300 novelty auditory oddball using HD-EEG (256 channel); *n* = 6 subgroup) NTB: MMSE, MoCA, NPI, activities of daily living.	Cognition: significant improvement in MMSE (versus nonsignificant decline in the wait-list group). Neuroimaging: significantly greater left temporal inferior gyrus volume. Significantly reduced P300 latency.	Single-blind, nonrandomised, waitlist-controlled pilot trial.

Yamaguchi et al. (2004) [30], Japan	Aim: to test the effects of choto-san on brain function in people with VaD/MCI. Recruitment location: hospital outpatients.	VaD/MCI (*N* = 20): choto-san (8M : 2F, 71.3 ± 9.8 yrs, MMSE 23.8 ± 3.6) or control (9M : 1F, 68.0 ± 8.6 yrs, MMSE 26.8 ± 2.8).	Intervention: 3x choto-san extract (TJ-47, Tsumura, 7.5 g/day) orally, daily [contains 4.5 g of extract of 11 kinds of dried medical herbs: Uncariae Uncis Cum Ramulus (3 g hooks and branch of *Uncaria sinensis* Oliver), Aurantii Nobilis pericarpium (3 g peel of *Citrus unshiu* Markovich), Pinelliae tuber (3 g tuber of *Pinellia ternate* Breitenbach), Ophiopgonis tuber (3 g root of *Ophiopogon japonicus* Ker-Gawler), Hoelen (3 g fungus of *Poria cocos* Wolf), Ginseng radix (2 g root of *Panax ginseng* C.A. Meyer), Chrysanthemi flow (2 g flower of *Chrysanthemum morifolium* Ramatulle), Saphoshnikoviae radix (2 g root and rhizome of *Saposhnikovia divaricata* Schischkin), Glycyrrhizae radix (1 g root of *Glycyrrhiza uralensis* Fisher), Gypsum Fibrosum (5 g CaSO_4_ 2H_2_O) and Zingiberis rhizoma (1 g, rhizome of *Zingiber officinale *Roscoe)]. Duration: 12 weeks.	ERP (P300 novelty auditory oddball) NTB: MMSE, verbal fluency test.	Cognition: significantly faster RT and increased accuracy on auditory oddball task. Significant improvement in MMSE and verbal fluency. Neuroimaging: Significant reduction in P300 latency. Trend towards increased novelty P300 amplitude.	Open, cohort study.

Zhang et al. (2015) [31], China	Aim: to investigate the effect of Bushen capsule (BSC) on brain function in people with aMCI. Recruitment location: hospital and community.	aMCI (*N* = 44): randomised to BSC (12M : 10F, 65.05 ± 6.67 yrs, MMSE 26.27 ± 1.58) or placebo (11M : 11F, 62.41 ± 5.76 yrs, MMSE 26.45 ± 1.40).	Intervention: 4 × 300 mg BSC [main components Zexie (*Alismatis rhizoma*) and Roucongrong (*Cistanches Herba*)] or 4x placebo tablet, 3x/day. Duration: 3 months.	fMRI (episodic memory encoding task). NTB: MMSE, AVLT, CVLT, Stroop, digit symbol, clock drawing.	Cognition: significant improvement in MMSE, stroop, and AVLT. Neuroimaging: increased brain activation in right putamen; this was significantly associated with stroop performance. Reduced brain deactivation in right middle temporal gyrus; this was significantly associated with AVLT performance. Adverse effects: 1x decreased appetite for 3 days (BSC), 1x mild nausea for 1 week (placebo). Neither discontinued use or withdrew from study.	Randomised, double-blind, placebo-controlled trial

Zhang et al. (2014) [32], China	Aim: to investigate the effect of Congrongyizhi capsule (CCRC) on brain function in people with aMCI. Recruitment location: hospital and community.	aMCI (*N* = 41): randomised to CCRC (8M : 8F, 64.25 ± 7.10 yrs, MMSE 26.38 ± 1.50), placebo (4M : 6F, 60.20 ± 3.52 yrs, MMSE 26.70 ± 1.64), or control (6M : 7F, 60.08 ± 6.53 yrs, MMSE 26.77 ± 1.30).	Intervention: 4x CCRC [main components Cistanche and Polygonum multiflorum thunb] or 4x placebo tablet, 3x/day or nothing (control). Duration: 3 months.	fMRI (n-back task). NTB: MMSE, AVLT, CVLT, stroop, digit span, clock drawing.	Cognition: significant improvement in MMSE and digit span, which were significantly associated with increased brain deactivation in posterior cingulate cortex. Neuroimaging: increased brain deactivation in posterior cingulate cortex, inferior frontal gyrus, and lingual gyrus. Adverse effects: 1x decreased appetite for 3 days (CCRC), 1x mild nausea for 1 week (placebo). Neither discontinued use or withdrew from study.	Randomised, double-blind, placebo-controlled trial.

Zhang et al. (2012) [33], China	Aim: to investigate the effect of *Ginkgo biloba* (EGb761) on brain activity in people with VaD. Recruitment location: hospital.	VaD (*N* = 80: 46M : 34F, 66.5 ± 5.6 yrs) randomised to GBT (*n* = 40) or aspirin (*n* = 40).	Intervention: 19.2 mg GBT + 75 mg aspirin or 75 mg aspirin tablet, 3x/day. Duration: 3 months.	rCBF (transcranial Doppler) NBT: Montreal cognitive assessment (MoCA).	Cognition: significant improvement in global score MoCA, as well as MoCA score indices for executive function, attention, delayed memory, and orientation. Neuroimaging: significant increase in blood flow velocity in middle and anterior cerebral arteries.	Randomised, controlled trial.

*Note*. ACC = anterior cingulate cortex; AD = Alzheimer's disease; ADAS-Cog = Alzheimer's Disease Assessment Scale–cognitive subscale; aMCI = amnestic mild cognitive impairment; AVLT = Auditory Verbal Learning Test; BNT = Boston Naming Test; CGI = clinical global impression; COWAT = Controlled Oral Word Association Test; CVLT = California Verbal Learning Test; d = day (i.e., /d = per day); DMN = default mode networks; DRS = Dementia Rating Scale; EEG = electroencephalography; ERP = event-related potential; FAB = frontal assessment battery; F : M = females to males; fMRI = functional magnetic resonance imaging; GM = grey matter; HVLT-R = Hopkins Verbal Learning Test-Revised; MCI = mild cognitive impairment; MFG = medial frontal gyrus; MID = multi-infarct dementia; MMSE = Mini-Mental State Examination; MoCA = Montreal cognitive assessment; MRI = magnetic resonance imaging; MTG = medial tegmental gyrus; NPI = Neuropsychiatric Inventory; NTB = neuropsychological test battery; OBS = Organic Brain Syndrome Scale; PCC = posterior cingulate cortex; PET = positron emission tomography; PSMS = Physical Self-Maintenance Scale; RAVLT = Rey Auditory Verbal Learning Test; rCBF = regional cerebral blood flow; RCFT = Rey Complex Figure Test; rCMRglc = regional cerebral metabolic rate of glucose consumption; ReHo = regional homogeneity; RT = reaction time; SCAG = Sandoz Clinical Assessment-Geriatric; SDAT = senile dementia of the Alzheimer type; SH = subcortical/periventricular hypertensity; SPECT = single-photon emission computed tomography; tHcy = total homocysteine; TMT = Trail Making Test; VaD = vascular dementia; V-EEG = vigilance-controlled EEG; yrs = years (i.e., age in years); WAIS-R = Wechsler Adult Intelligence Scale–Revised; WMS-R = Wechsler Memory Scale–Revised.

**Table 4:** Risk of bias ratings for included studies. Studies are detailed in alphabetical order of authors' names. Studies with low risk of bias (total scores ≥ 9) are italicised.

Study	Item 1	Item 2	Item 3	Item 4	Item 5	Item 6	Item 7	Item 8	Item 9	Item 10	Total
Bi et al. (2011)	0	0	1	0	1	1	1	1	0	0	5
Cohen et al. (2003)	0	0	1	0	1	1	1	1	0	0	5
*de Waal et al. (2014)*	*1*	*1*	*1*	*1*	*1*	*1*	*1*	*1*	*1*	*1*	*10*
*Douaud et al. (2013)*	*1*	*1*	*1*	*1*	*1*	*1*	*1*	*1*	*1*	*0*	*9*
Heo et al. (2016)	0	0	1	0	0	0	0	1	1	0	3
Hofferberth (1994)	0	0	0	0	0	0	0	1	0	0	1
*Jernerén et al. (2015)*	*1*	*1*	*1*	*1*	*1*	*1*	*1*	*1*	*1*	*0*	*9*
Köbe et al. (2015)	0	0	1	0	0	1	1	1	1	0	5
Matsuoka et al. (2012)	0	0	1	0	1	1	1	1	1	0	6
Muresanu et al. (2010)	0	0	1	0	0	0	1	1	0	0	3
Nilsson et al. (2000)	0	0	1	0	1	1	0	1	0	0	4
Oishi et al. (1998)	0	0	0	0	0	0	0	1	1	0	2
Onofrj et al. (2002)	0	0	1	1	1	0	0	1	1	0	5
Saletu et al. (1995)	0	0	1	0	1	0	1	1	1	1	6
*Smith et al. (2010)*	*1*	*1*	*1*	*1*	*1*	*1*	*1*	*1*	*1*	*1*	*10*
Thomas et al. (2001)	1	0	0	0	1	1	1	1	0	0	5
Tsolaki et al. (in press)	0	0	0	0	0	0	0	0	0	0	1
Yamaguchi et al. (2004)	0	0	0	0	0	0	0	1	0	0	1
Zhang et al. (2015)	0	0	1	1	0	1	1	1	1	0	6
Zhang et al. (2014)	0	0	1	1	0	1	1	1	1	0	6
Zhang et al. (2012)	1	0	1	0	1	0	0	1	0	0	4

*Note. *Items rated as yes scored 1, and items rated as no or unable to determine both scored 0. Lower scores indicate a higher risk of bias.
