# Supplementary material for: A Systematic Review of Intervention Studies Examining Nutritional and Herbal Therapies for Mild Cognitive Impairment and Dementia Using Neuroimaging Methods: Study Characteristics and Intervention Efficacy
Source: Evid Based Complement Alternat Med. 2017 Feb 19;2017:6083629. doi: 10.1155/2017/6083629 (PMC5337797; doi:10.1155/2017/6083629)
Supplement: Supplementary file 1 — Table S1. Keywords and example search strategy used in Scopus. [file 6083629.f1.docx]

| *Table S1.* Keywords and example search strategy used in Scopus. | |
| --- | --- |
| **Searches** | **Strings** |
| Population | dementia OR "vascular dementia" OR alzheimer* OR "older adult*" OR aging OR ageing OR "mild cognitive impairment" OR "early onset" OR “age associated cognitive decline” OR “age related cognitive decline” OR “age associated memory impairment” OR “cognitive decline” |
| Intervention | "herbal medicine*" OR qigong OR yoga OR “tai chi” OR acupuncture OR "control* trial" OR "Chinese medicine*" OR "complementary medicine*" OR "alternative medicine*" OR "natural medicine*" OR vitamin* OR nutraceutical* OR "nutritional supplement*" OR “Chinese herbal medicine” OR “traditional Chinese medicine” OR nootropic* OR curcumin OR ginkgo |
| Outcome | neuroimaging OR MRI OR fMRI OR electroencephalogra* OR EEG OR “event related potential*” OR ERP* OR MEG OR SSEP* OR NIRS OR PET OR "Simultaneous EEG/fMRI" OR "cerebral blood flow" OR CBF OR “computed tomography” OR SPECT or “magnetic resonance spectroscopy” OR MRS |
| Scopus Search: | Population AND Intervention AND Outcome |
